# The relationship between preoperative anxiety, self-efficacy, and positive coping style in lung cancer patients undergoing video-assisted thoracoscopic surgery: a cross-sectional study

**DOI:** 10.1186/s40359-026-05324-5

**Published:** 2026-08-10

**Authors:** Shuai Zhang, Liurong Cheng, Honghua Guo, Zi’ping Wang, Yili Li, lu Qiao, Andie Chen, Chuxia Lai, Jiaqi Li, Xing Gao, Zhaohao He

**Affiliations:** 1 Nursing Department, Hainan General Hospital (Hainan Affiliated Hospital of Hainan Medical University ), Haikou, Hainan China; 2 Department of the Operation room, Hainan General Hospital (Hainan Affiliated Hospital of Hainan Medical University ), Haikou, Hainan China; 3 Nursing School, Hainan Medical University, Haikou, Hainan Province China; 4 Thoracic Department, Hainan General Hospital (Hainan Affiliated Hospital of Hainan Medical University ), Haikou, Hainan China; 5 School of Clinical Medicine, Hainan Medical University, Haikou, Hainan Province China

**Keywords:** Lung cancer, Preoperative anxiety, Self-efficacy, Positive coping style, Mediating effect

## Abstract

**Background:**

Patients with lung cancer undergoing video-assisted thoracoscopic surgery (VATS) may experience preoperative anxiety arising from cancer-related uncertainty and surgery-related concerns. Although VATS is less invasive than conventional thoracotomy, patients may still worry about malignancy, prognosis, anaesthesia, postoperative recovery, and long-term health. General self-efficacy and positive coping style are potentially modifiable psychological resources associated with preoperative anxiety, but their interrelationships and the potential indirect role of positive coping style remain insufficiently understood.

**Materials and methods:**

This cross-sectional study recruited patients with lung cancer scheduled for VATS from a large tertiary grade-A general hospital in Hainan Province, China, between April 2023 and October 2024. Preoperative anxiety, general self-efficacy, and positive coping style were assessed using validated questionnaires. A total of 387 valid questionnaires were analysed. Descriptive statistics, group comparisons, Spearman’s correlation analysis, structural equation modelling, and Bootstrap mediation analysis were performed using SPSS 26.0 and AMOS.

**Results:**

A total of 387 valid questionnaires were included in the analysis, yielding a valid response rate of 89.38%. The mean scores of preoperative anxiety, general self-efficacy, and positive coping style were 34.88 ± 13.54 out of 64, 22.21 ± 5.95 out of 40, and 19.57 ± 7.30 out of 36, respectively, with corresponding standardized scores of 54.50%, 55.53%, and 54.36%. Spearman’s correlation analysis showed that preoperative anxiety was negatively correlated with general self-efficacy (r=-0.384, P < 0.01) and positive coping style (r=-0.415, P < 0.01), while general self-efficacy was positively correlated with positive coping style (r = 0.642, P < 0.01). In the structural equation model, general self-efficacy was positively associated with positive coping style (β = 0.795, P < 0.001), and positive coping style was negatively associated with preoperative anxiety (β=-0.294, P = 0.006). The direct path from general self-efficacy to preoperative anxiety was not statistically significant (β=-0.154, P = 0.131).

**Conclusion:**

This cross-sectional study documented preoperative anxiety among patients with lung cancer awaiting VATS, together with relatively low levels of general self-efficacy and positive coping style. The observed indirect-only statistical mediation pattern suggests that general self-efficacy was indirectly associated with lower preoperative anxiety through positive coping style. This pattern extends the understanding of psychological factors associated with preoperative anxiety and may inform combined assessment of anxiety, self-efficacy, and coping responses, as well as targeted preoperative support aimed at strengthening patients’ confidence, improving their understanding of perioperative information, and encouraging positive coping.

**Clinical trial number:**

Not applicable.

**Supplementary Information:**

The online version contains supplementary material available at https://doi.org/10.1186/s40359-026-05324-5.

## Background

Lung cancer remains a major cause of cancer-related morbidity and mortality worldwide and in China [1, 2]. Video-assisted thoracoscopic surgery (VATS) has become a widely used surgical approach because it is associated with less surgical trauma, less postoperative pain and faster recovery than conventional thoracotomy [3, 4]. Despite these advantages, patients awaiting VATS may experience anxiety arising from both cancer-related uncertainty and surgery-related concerns. Questions about diagnosis, disease stage and prognosis may coexist with worries about anaesthesia, postoperative discomfort, chest tube management and recovery [5]. Previous studies have reported that preoperative anxiety is common among patients with lung cancer undergoing VATS, with prevalence estimates ranging from 25% to 70%. Sex, information needs, illness perception and patient–physician trust have been associated with its severity [4, 6, 7]. Its reported associations with perioperative and postoperative outcomes further underscore the clinical importance of recognising and addressing anxiety before surgery [8–10].

General self-efficacy and positive coping style are potentially modifiable psychological resources that may be relevant to preoperative anxiety. Self-efficacy refers to an individual’s belief in their ability to organise and perform the actions needed to manage a particular situation [11]. Patients with stronger self-efficacy may feel more capable of understanding perioperative information, communicating with healthcare professionals and managing surgery-related stress [12, 13]. Positive coping involves active efforts to obtain information, solve problems, seek support and regulate distress [14]. Previous research has linked coping style with psychological adjustment and quality of life among patients with cancer [15, 16]. However, little is known about how self-efficacy and positive coping operate together in patients with lung cancer awaiting VATS.

Social Learning Theory provides a framework for examining these relationships [17]. The theory proposes that beliefs about personal capability influence how individuals appraise stressful situations and the actions they take in response [18]. In the preoperative setting, patients with stronger self-efficacy may be more likely to adopt positive coping strategies, which may in turn be associated with lower anxiety. Positive coping may therefore represent a behavioural pathway linking patients’ confidence with their emotional response to surgery. This pathway has not been adequately examined in patients with lung cancer undergoing VATS.

This study investigated preoperative anxiety, general self-efficacy and positive coping style among patients with lung cancer awaiting VATS and examined whether positive coping statistically mediated the association between self-efficacy and anxiety. We hypothesised that general self-efficacy would be positively associated with positive coping and negatively associated with preoperative anxiety, that positive coping would be negatively associated with preoperative anxiety, and that positive coping would mediate the association between general self-efficacy and preoperative anxiety.

## Methods study design

This study was a cross-sectional design and prepared following the guidelines set out in the STROBE statement, to ensure transparency and quality of results [19].

### Participants and setting

A convenience sampling method was used to recruit patients with lung cancer from a large tertiary grade-A general hospital in Hainan Province, China, between April 2023 and October 2024. In China, tertiary grade-A hospitals represent the highest category in the national hospital classification system and are evaluated based on medical capacity, clinical quality, management, facilities, and service standards. The thoracic surgery department has 57 beds and performs approximately 1,500 thoracic surgical procedures annually, including lung, oesophageal, and mediastinal procedures, of which more than 1,000 are minimally invasive lung-related procedures. These figures were provided only to describe the clinical service capacity and case availability of the department and were not used as the sampling denominator for the present study.

During the study period, 1,650 thoracic surgical procedures were recorded in the department’s surgical registry, including 1,134 thoracoscopic lung-related procedures. Among these, 558 patients underwent VATS for lung cancer and were screened for eligibility in this study. Of these 558 patients, 125 were not enrolled for reasons related to eligibility, refusal to participate, or inability to complete the questionnaire; detailed counts for each specific reason were not separately recorded during recruitment. A total of 433 eligible and accessible patients completed the questionnaire. After questionnaire screening, 46 questionnaires were excluded from the final analysis, including 45 with missing data and 1 with an obvious response pattern in which most items were answered using the same option. Therefore, 387 valid questionnaires were included in the analysis, yielding a valid response rate of 89.38%.

The inclusion criteria were as follows: (1) diagnosed with or clinically suspected of non-small cell lung cancer based on clinical, imaging, and/or pathological findings and scheduled for video-assisted thoracoscopic surgery; (2) classified as American Society of Anesthesiologists (ASA) grade I or II; (3) aged ≥ 18 years; and (4) able to understand and complete the questionnaire.

The exclusion criteria were as follows: (1) a history of diagnosed mental illness; (2) cognitive impairment or impaired consciousness that prevented questionnaire completion; and (3) severe hearing, visual, language, or communication impairment affecting questionnaire responses. All patients who completed the questionnaire provided written informed consent.

### Sample size calculation

Sample size was calculated using the formula for cross-sectional studies: n=(Z_1−α/2_ /δ)^2^×*p*×(1 − *p*) where Z_1−α/2_=1.96 when α = 0.05, the expected prevalence of preoperative anxiety among patients with lung cancer was 25%–70%, and δ is admissible error (which was 5% here) [4]. The theoretical sample size calculated was 317 ~ 423, inclusive of an additional 10% to accommodate potential subject loss during the course of the study (the assumed degree of variability of *p* = 0.5 maximizes the required sample size).

### Questionnaire introduction

The questionnaire consisted of four parts. Part 1 was a general information questionnaire developed by the research team, which included 12 items: sex, age, ethnicity, marital status, religious belief, educational level, place of residence, occupation, employment status, history of surgery, method of medical expense payment, and monthly income.

The Preoperative Anxiety Scale was originally developed by Burton et al. [20] and subsequently adapted into Chinese by Li Rui et al. [21], which was used to assess the level of preoperative anxiety in patients. The Cronbach’s α coefficient of the scale was 0.934, and the Cronbach’s α coefficients for its dimensions ranged from 0.835 to 0.863. The content validity index was 0.980, and the Kaiser-Meyer-Olkin (KMO) value was 0.925. The scale consists of 16 items across three dimensions: concerns about health (6 items), concerns about recovery (5 items), and concerns about the surgical process (5 items). Each item is scored on a 5-point Likert scale (never = 0, rarely = 1, occasionally = 2, often = 3, always = 4), with a total possible score ranging from 0 to 64. Higher scores indicate more severe anxiety.

The General Self-Efficacy Scale was used to assess general self-efficacy in patients with lung cancer. The scale was originally developed by Ralf Schwarzer et al. [18]and subsequently revised by Wang Caikang et al. [22]. It consists of 10 items, each scored on a 4-point Likert scale ranging from 1 to 4 (1 = not at all true, 2 = barely true, 3 = moderately true, 4 = exactly true). The total score ranges from 10 to 40, with higher scores indicating stronger self-efficacy. The Cronbach’s α coefficient of the scale was 0.83 [23].

The Simplified Coping Style Questionnaire originally consisted of two dimensions: positive coping (12 items) and negative coping (8 items). The questionnaire was developed by Folkman S et al. [24] and subsequently adapted into Chinese by Xie Yaning et al. [25]. In this study, only the positive coping dimension was used. Each item is scored on a 4-point scale (0 = never, 1 = occasionally, 2 = sometimes, 3 = often), with a total score ranging from 0 to 36. Higher scores indicate a greater tendency for patients to adopt positive coping styles. The Cronbach’s α coefficient for the positive coping dimension was 0.89. This scale has been widely used in research and demonstrates good reliability and validity.

### Data collection

Prior to the survey, all preoperative visit nurses received standardized training using a unified protocol, and a pilot survey was conducted to ensure that the questionnaire items were unambiguous and easy to understand, thereby improving the quality of the survey. On the day before surgery, the survey was administered using paper-based questionnaires distributed on site. The visiting nurses explained the purpose, methods, and significance of the survey, and the questionnaires were completed by the patients with lung cancer or with assistance from their family members.

### Quality control

Questionnaires were completed and collected on site. After completion, the survey nurses checked each questionnaire on the spot and, if any items were missing, reminded the participants to fill them in immediately. Questionnaires with obviously inconsistent responses or with all items answered using the same option were excluded.

### Ethical considerations

This study was approved by the Medical Ethics Committee of Hainan General Hospital before data collection began (Approval No. Yi Lun Yan [2023] No. 361; approval date: 1 March 2023). Data were collected between April 2023 and October 2024. All participants provided written informed consent before completing the questionnaire. All procedures were conducted in accordance with the approved ethical protocol and the Declaration of Helsinki.

### Statistical analysis

All data were imported into Microsoft Excel and independently verified by two researchers to ensure accuracy. Statistical analyses were performed using IBM SPSS Statistics version 26.0 (IBM Corporation, Armonk, NY, USA). Continuous variables were first assessed for normality using the Shapiro–Wilk test. Variables with a normal distribution were described using means and standard deviations (SD), whereas non-normally distributed variables were summarized using medians and interquartile ranges (IQR). Categorical variables were presented as frequencies and percentages.

Based on the nature and distribution of the data, independent-samples *t* tests, analysis of variance (ANOVA), and rank-sum tests were applied, as appropriate. Associations among preoperative anxiety, general self-efficacy, and positive coping style were examined using Spearman’s correlation analysis. Correlation coefficients were interpreted as follows: |r| ≥ 0.70 indicated a strong correlation, 0.30 ≤ |r| < 0.70 indicated a moderate correlation, and |r| < 0.30 indicated a weak correlation [26].

Mediation analysis was conducted using IBM SPSS Amos to examine the relationships among preoperative anxiety, general self-efficacy, and positive coping style. The mediation model was constructed based on previous literature [23], Social Learning Theory [17], clinical relevance, socioeconomic relevance, and the results of univariate analyses. Place of residence, method of medical payment, monthly income, and education level were included as covariates because they were significantly associated with preoperative anxiety in the univariate analysis and may also influence patients’ access to medical resources, financial burden, health literacy, understanding of perioperative information, risk perception, and coping behaviours. These covariates were included to reduce potential confounding when examining the associations among general self-efficacy, positive coping style, and preoperative anxiety. The mediating effect was tested using bootstrapping with 5,000 resamples. A two-sided *P* value < 0.05 was considered statistically significant.

## Results

### Demographic characteristics

A total of 433 patients with lung cancer were surveyed. After excluding 46 invalid questionnaires, 387 valid questionnaires were included in the analysis, yielding a valid response rate of 89.38%. More than two-thirds of lung cancer patients are aged 50 years or older. The numbers of male and female patients were similar. The majority of patients were married (86.3%), of Han ethnicity (91.5%), and without religious belief (94.6%). More than half of the patients had an education level of junior high school or below. Rural patients outnumbered urban patients (58.9% vs. 41.1%). The proportion of patients with a history of surgery was slightly lower than that without (46.3% vs. 53.7%). More than one-third of the patients had a monthly income of less than 1,500 RMB. Half of the patients covered their medical expenses through the New Rural Cooperative Medical Scheme, and most patients were employed (48.3%). Detailed demographic characteristics are presented in Table 1.

**Table 1:** Comparison of general characteristics and preoperative anxiety scores among patients with lung cancer undergoing VATS

Variable	*n* (%)	Score (mean ± SD)	t/F	*P*
Age (years)			12.690	< 0.001
18–29	18 (4.6)	42.89 ± 12.33		
30–39	18 (4.6)	44.17 ± 7.80		
40–49	56 (14.6)	38.11 ± 13.27		
≥ 50	295 (76.2)	33.21 ± 13.47		
Sex			-5.150	< 0.001
Male	197 (50.9)	31.52 ± 14.05		
Female	190 (49.1)	38.37 ± 12.08		
Marital status			44.396	< 0.001
Unmarried	19 (4.9)	41.53 ± 13.01		
Married	334 (86.3)	33.84 ± 13.35		
Divorced	14 (3.6)	47.93 ± 3.58		
Widowed	20 (5.2)	36.90 ± 15.50		
Ethnicity			-0.079	0.937
Han	354 (91.5)	34.86 ± 13.44		
Minority	33 (8.5)	35.06 ± 14.84		
Religious belief			-1.158	0.258
Yes	21 (5.4)	32.33 ± 10.15		
No	366 (94.6)	35.03 ± 13.71		
Education level			3.338	0.022
Junior high school or below	212 (54.8)	36.17 ± 12.30		
Technical secondary school or high school	96 (24.8)	33.33 ± 15.32		
Associate degree	43 (11.1)	35.72 ± 16.56		
Bachelor’s degree or over	36 (9.3)	30.42 ± 10.31		
Residence			3.691	< 0.001
Rural	228 (58.9)	37.00 ± 12.84		
Urban	159 (41.1)	31.84 ± 13.98		
Occupation			6.938	< 0.001
Farmer	164 (42.4)	37.41 ± 11.65		
Worker	94 (24.3)	36.10 ± 13.84		
Civil servant	21 (5.4)	28.00 ± 11.83		
Freelance	108 (27.9)	31.32 ± 15.14		
Employment status			18.726	< 0.001
Retired	106 (27.4)	28.08 ± 13.71		
Unemployed	94 (24.3)	37.49 ± 11.74		
Other	187 (48.3)	37.42 ± 13.01		
Medical expense payment method			14.754	< 0.001
Urban medical insurance	178 (46.0)	31.04 ± 13.76		
New Rural Cooperative Medical Scheme	194 (50.1)	38.38 ± 11.88		
Self-pay or Commercial insurance	15(3.9)	35.13 ± 18.92		
Monthly income (RMB)			22.388	< 0.001
< 1500	140 (36.1)	39.19 ± 11.72		
1500–2999	121 (31.3)	36.48 ± 12.59		
3000–4999	54 (14.0)	32.69 ± 15.78		
≥ 5000	72 (18.6)	25.46 ± 11.81		

*VATS* video-assisted thoracoscopic surgery, *SD* standard deviation, *RMB* Renminbi

P values < 0.001 are presented as < 0.001

In this study, the mean scores of preoperative anxiety, general self-efficacy, and positive coping style among patients with lung cancer undergoing VATS were 34.88 ± 13.54, 22.21 ± 5.95, and 19.57 ± 7.30, respectively. The corresponding standardized scores were 54.50%, 55.53%, and 54.36%, respectively. Spearman’s correlation analysis showed that preoperative anxiety was negatively correlated with general self-efficacy (*r*=-0.384, *P* < 0.01) and positive coping style (*r*=-0.415, *P* < 0.01). In addition, general self-efficacy was positively correlated with positive coping style (*r* = 0.642, *P* < 0.01). Detailed results are presented in Tables 2 and 3.

**Table 2:** Scores of preoperative anxiety, General self-efficacy, positive coping style

Dimension	Number of items	Score range	Score (mean ± SD)	Standardized score
Preoperative anxiety	16	0 ~ 64	34.88 ± 13.54	54.50
General Self-efficacy	10	10 ~ 40	22.21 ± 5.95	55.53
Positive coping style	12	0 ~ 36	19.57 ± 7.30	54.36

Score rate = (mean score / maximum possible score) × 100%. A score rate of < 60% on the total scale or any subscale indicates poor, 60%–85% indicates moderate, and > 85% indicates good [27]

**Table 3:** Spearman correlations among preoperative anxiety, general self-efficacy, and positive coping style in lung cancer patients undergoing VATS

Item	Preoperative anxiety	General Self-efficacy	Positive coping style
Preoperative anxiety	1		
General Self-efficacy	-0.384^**^	1	
Positive coping style	-0.415^**^	0.642^**^	1

** At a confidence level (two-tailed) of 0.01, the correlation is significant

### Mediation analysis

**Fig. 1:**
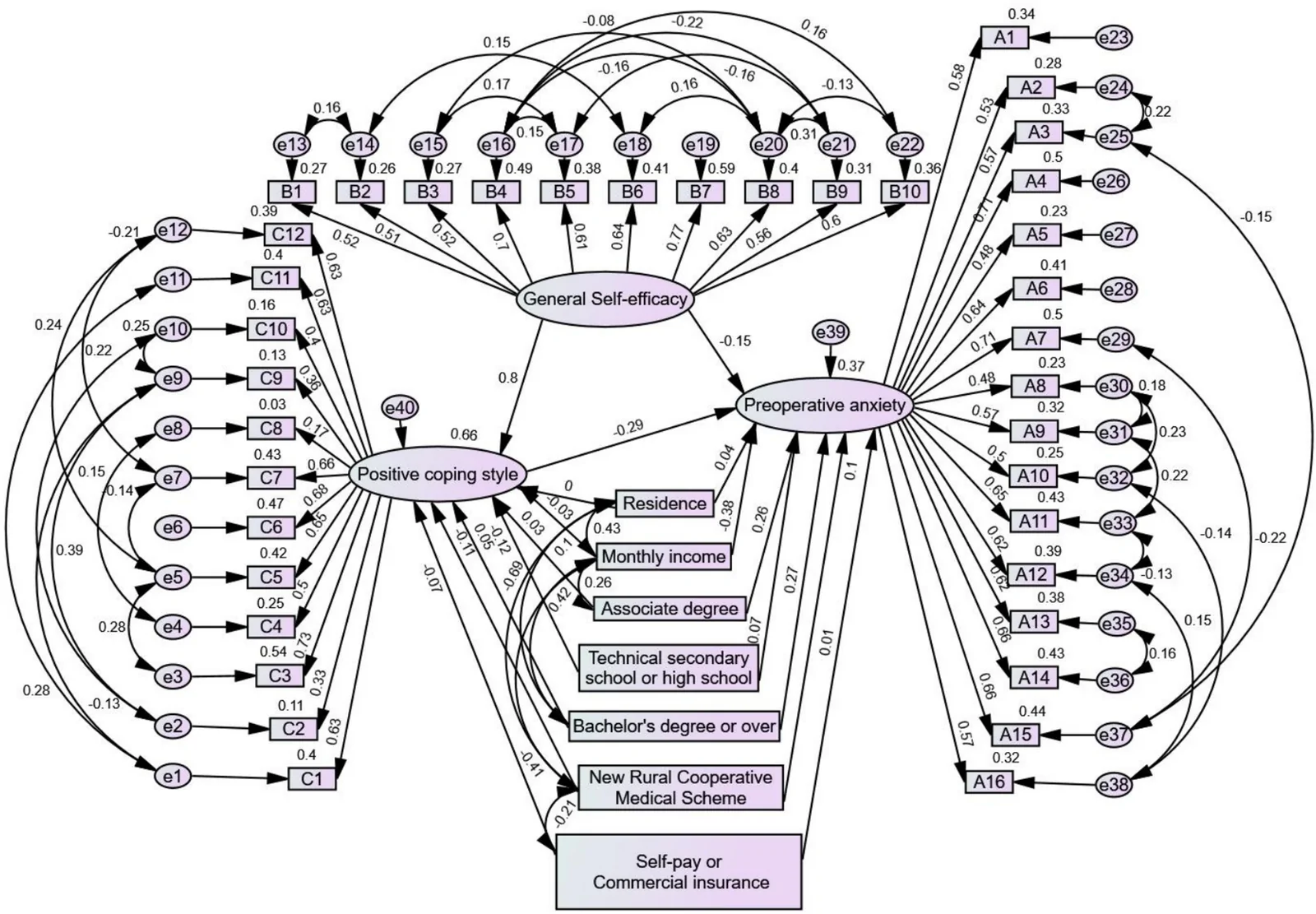
Standardized path diagram for the mediating effect

As shown in Fig. 1, the structural equation model was estimated to examine the relationships among general self-efficacy, positive coping style, and preoperative anxiety, with place of residence, method of medical payment, monthly income, and education level included as covariates. The chi-square-to-degrees-of-freedom ratio (CMIN/DF) was 1.933, and the root mean square error of approximation (RMSEA) was 0.049. The incremental fit indices were IFI = 0.870, TLI = 0.855, and CFI = 0.868. Although the CMIN/DF and RMSEA indicated a good absolute fit, the IFI, TLI, and CFI remained below the conventional threshold of 0.90, indicating mixed model-fit evidence. Overall, the model fit was considered marginally acceptable.

As shown in Table 4, general self-efficacy was positively associated with positive coping style (standardised coefficient β = 0.795, *P* < 0.001). Positive coping style was negatively associated with preoperative anxiety (β = −0.294, *P* = 0.006), whereas the direct association between general self-efficacy and preoperative anxiety was not statistically significant (β = −0.154, *P* = 0.131).

**Table 4:** Structural Equation Model Path Analysis Results

Path	Standardized coefficient	Estimate	S.E.	C.*R*.	*P*	Bootstrap95% CI		Bias-corrected bootstrap 95% CI	
						Lower	Upper	Lower	Upper
Positive coping style ← General Self-efficacy	0.795	1.248	0.151	8.254	< 0.001	0.961	1.641	0.958	1.634
Positive coping style ← Place of residence	0.001	0.001	0.080	−0.003	0.993	−0.165	0.167	−0.174	0.154
Positive coping style ← Associate degree	0.030	0.065	0.091	0.716	0.474	−0.168	0.305	−0.167	0.305
Positive coping style ← Secondary school or high school	−0.117	−0.186	0.063	−2.925	0.003	−0.341	−0.025	−0.348	−0.033
Positive coping style ← Bachelor’s degree or above	0.048	0.112	0.106	1.058	0.290	−0.138	0.345	−0.148	0.340
Positive coping style ← New Rural Cooperative Medical Scheme	−0.114	−0.156	0.081	−1.921	0.055	−0.318	−0.009	−0.309	−0.003
Positive coping style ← Self-payment or commercial insurance	−0.074	−0.261	0.147	−1.777	0.076	−0.715	0.182	−0.703	0.203
Positive coping style ← Monthly income	−0.030	−0.020	0.035	−0.563	0.573	−0.100	0.052	−0.092	0.063
Preoperative anxiety ← Positive coping style	−0.294	−0.327	0.118	−2.762	0.006	−0.62	−0.073	−0.615	−0.067
Preoperative anxiety ← General Self-efficacy	−0.154	−0.269	0.178	−1.509	0.131	−0.724	0.174	−0.734	0.147
Preoperative anxiety ← Place of residence	0.042	0.067	0.100	0.668	0.504	−0.172	0.298	−0.175	0.297
Preoperative anxiety ← Associate degree	0.257	0.619	0.122	5.071	< 0.001	0.335	0.894	0.346	0.902
Preoperative anxiety ← Secondary school or high school	0.065	0.115	0.081	1.42	0.156	−0.114	0.332	−0.101	0.344
Preoperative anxiety ← Bachelor’s degree or above	0.267	0.696	0.142	4.907	< 0.001	0.388	1.011	0.397	1.021
Preoperative anxiety ← New Rural Cooperative Medical Scheme	0.098	0.149	0.102	1.453	0.146	−0.074	0.374	−0.072	0.374
Preoperative anxiety ← Self-payment or commercial insurance	0.008	0.031	0.184	0.166	0.868	−0.483	0.509	−0.442	0.550
Preoperative anxiety ← Monthly income	−0.384	−0.281	0.048	−5.869	< 0.001	−0.385	−0.179	−0.384	−0.178

Place of residence was coded as 0 = rural and 1 = urban, with rural residence as the reference category. For method of medical payment, urban medical insurance was used as the reference category, and two dummy variables were created for the New Rural Cooperative Medical Scheme and self-payment/commercial insurance. For education level, junior high school or below was used as the reference category, and three dummy variables were created for technical secondary school or high school, associate degree, and bachelor’s degree or above. Monthly income was treated as an ordinal variable and coded as 1 = < 1,500 RMB, 2 = 1,500–2,999 RMB, 3 = 3,000–4,999 RMB, and 4 = ≥ 5,000 RMB

*SE* standard error, *CR* critical ratio, *CI* confidence interval

After adjustment for the covariates, participants with an associate degree (β = 0.257, *P* < 0.001) or a bachelor’s degree or above (β = 0.267, *P* < 0.001) had higher preoperative anxiety than those with a junior high school education or below. No significant difference was observed for participants with a technical secondary school or high school education (β = 0.065, *P* = 0.156). Higher monthly income was associated with lower preoperative anxiety (β = −0.384, *P* < 0.001). Place of residence and the two medical payment contrasts were not significantly associated with preoperative anxiety.

For positive coping style, participants with a technical secondary school or high school education had a lower score than those with a junior high school education or below (β = −0.117, *P* = 0.003). Compared with participants covered by urban medical insurance, those covered by the New Rural Cooperative Medical Scheme also had a lower positive coping style score (β = −0.114; unstandardised estimate = − 0.156; bias-corrected bootstrap 95% CI: −0.309 to − 0.003). Although the corresponding Wald test was marginal (*P* = 0.055), the bootstrap interval excluded zero. None of the remaining covariates was significantly associated with positive coping style.

The mediating effect of positive coping style on the association between general self-efficacy and preoperative anxiety was examined using 5,000 bootstrap resamples and bias-corrected 95% confidence intervals (CIs) (Table 5). The indirect effect was statistically significant (point estimate = − 0.408, bias-corrected 95% CI: −0.782 to − 0.094), accounting for 60% of the total effect. In contrast, the direct effect was not statistically significant (point estimate = − 0.269, bias-corrected 95% CI: −0.734 to 0.147). The total effect remained statistically significant (point estimate = − 0.676, bias-corrected 95% CI: −0.984 to − 0.452). These findings were consistent with an indirect-only statistical mediation pattern, in which general self-efficacy was indirectly associated with preoperative anxiety through positive coping style.

**Table 5:** Bootstrap Mediation Effect Test Results

Effect	Point Estimate	Coefficient Product		Bootstrap95%CI		Bootstrap95%CI bias-corrected CI		SREP
		SE	Z	LLV	ULV	LLV	ULV	
Indirect Effect	-0.408	0.177	-2.305	-0.775	-0.092	-0.782	-0.094	60%
Direct Effect	-0.269	0.222	-1.212	-0.724	0.174	-0.734	0.147	40%
Total Effect	-0.676	0.136	-4.971	-0.974	-0.447	-0.984	-0.452	

*ULV* is Upper limit value, *LLV* is lower limiting value, *SREP* is standardized relative effect proportion

## Discussion

This study identified positive coping as a potential pathway linking general self-efficacy with preoperative anxiety among patients with lung cancer awaiting VATS. The findings partially supported our hypotheses and highlighted the importance of considering patients’ confidence and coping responses together rather than viewing preoperative anxiety as an isolated symptom.

Preoperative anxiety was prominent among patients with lung cancer awaiting VATS, with a higher level than that reported in previous studies of patients with lung cancer and general surgical populations [28, 29]. Patients preparing for lung cancer surgery face concerns about diagnosis and prognosis alongside uncertainty about anaesthesia, postoperative pain, chest tube management and recovery. Female patients reported higher anxiety, consistent with previous evidence of sex differences in preoperative and cancer-related psychological distress [30, 31].

Financial and educational circumstances may be relevant to preoperative anxiety. Higher monthly income was associated with lower anxiety after adjustment, possibly because limited financial resources increase concerns about treatment costs, income disruption and postoperative care, although financial toxicity was not directly assessed [32]. The association with education was more complex: participants with a bachelor’s degree or above had the lowest unadjusted anxiety score, whereas those with an associate degree or a bachelor’s degree or above had higher adjusted anxiety than participants with a junior high school education or below. This reversal suggests that education may operate through competing pathways and interact with broader socioeconomic circumstances. Greater educational attainment may facilitate understanding of perioperative information while also increasing awareness of surgical risks and prognostic uncertainty. Education should therefore not be regarded as a simple indicator of psychological preparedness; patients’ actual information needs and responses to risk should be assessed individually [33].

Positive coping may also vary according to patients’ social and resource contexts. Participants with a technical secondary school or high school education reported lower positive coping than those with a junior high school education or below. Those covered by the New Rural Cooperative Medical Scheme also tended to report lower positive coping than participants covered by urban medical insurance, although this finding requires cautious interpretation because the Wald test and bootstrap interval yielded marginally different evidence. Previous research has linked social support with self-efficacy and positive coping among Chinese patients with lung cancer [34], while the economic burden of treatment may persist despite medical insurance reimbursement [35]. These findings suggest that coping responses should be interpreted in relation to patients’ available social and practical resources rather than education or payment category alone.

Positive coping may represent a behavioural pathway through which confidence is translated into practical preparation. Patients may clarify unfamiliar aspects of the surgical pathway, practise recommended exercises, discuss specific concerns with healthcare professionals and involve family members when support is needed. These actions do not remove all uncertainty surrounding surgery, but they can turn diffuse concerns into manageable steps and give patients a greater sense of control before VATS.

General self-efficacy was positively associated with positive coping, which was in turn negatively associated with preoperative anxiety. Patients with stronger self-efficacy may perceive surgery-related demands as more manageable, regulate their emotions more effectively [36], and be more willing to seek information, prepare for surgery and obtain support [37]. The significant indirect effect, together with the non-significant direct path, suggests that positive coping may help explain how patients’ confidence is associated with their emotional adjustment before VATS. This interpretation is consistent with previous studies linking stronger self-efficacy with a more constructive outlook and greater use of support during illness-related stress [38, 39].

Social Learning Theory provides a framework for interpreting these associations. Mastery experience, vicarious learning, verbal encouragement and the interpretation of emotional responses are established sources of self-efficacy [40]. In preoperative care, these may be supported through achievable preparatory tasks, observation of others’ experiences, clear feedback and reassurance about anxiety. Such experiences may strengthen patients’ confidence and encourage positive coping, which may help them manage the emotional demands of impending surgery.

### Clinical implications

The findings suggest the value of assessing self-efficacy and coping responses alongside preoperative anxiety. During routine assessment, nurses could explore whether patients feel capable of managing surgery-related demands, understand the main stages of the VATS pathway and know how to obtain practical or emotional support. Additional support should be based on patients’ individual needs, such as low confidence, limited coping resources, difficulty understanding perioperative information or financial concerns, rather than on education or medical payment category alone.

Support could follow a structured sequence of explanation, demonstration, practice and feedback. Nurses can explain the main stages of VATS and postoperative recovery, demonstrate essential breathing and coughing exercises, and use teach-back to confirm understanding [41]. Helping patients complete achievable preparatory tasks and providing timely feedback may offer mastery experiences that strengthen self-efficacy. Nurses can also help patients clarify specific concerns, develop manageable preparation plans and seek support from healthcare professionals or family members. Patients experiencing persistent psychological distress or financial difficulty may be referred to psychological, medical insurance or social services as appropriate [42]. This approach addresses both self-efficacy and positive coping, which were central to the observed mediation pattern.

### Limitations

This study has several limitations. First, all variables were assessed using self-report questionnaires at a single time point, which may have introduced response and social desirability biases as well as common-method variance. Second, the cross-sectional design precludes conclusions about temporal ordering or causality; therefore, the observed mediation pattern should be interpreted as associative. Longitudinal and intervention studies are needed to examine whether changes in self-efficacy and positive coping precede changes in preoperative anxiety. Third, this single-centre study used convenience sampling, which may limit the representativeness and generalisability of the findings. The proportion of participants aged 18–49 years may also differ from that in the broader population of patients undergoing lung cancer surgery. Multicentre studies using consecutive or stratified sampling would provide more representative evidence. Fourth, clinical stage was not included because imaging-based staging was not recorded consistently before the survey and definitive pathological staging was generally unavailable until after surgery. Other potentially relevant clinical and psychosocial factors, including comorbidities, financial strain, health literacy and information needs, were also not directly assessed, leaving the possibility of residual confounding. Future studies should incorporate these factors to clarify the robustness of the observed associations. Nevertheless, the present findings provide preliminary evidence of an indirect association between general self-efficacy and preoperative anxiety through positive coping among patients with lung cancer awaiting VATS.

## Conclusion

This cross-sectional study documented preoperative anxiety among patients with lung cancer awaiting VATS, together with relatively low levels of general self-efficacy and positive coping style. The observed indirect-only statistical mediation pattern suggests that general self-efficacy was indirectly associated with lower preoperative anxiety through positive coping style. This pattern extends the understanding of psychological factors associated with preoperative anxiety and may inform combined assessment of anxiety, self-efficacy, and coping responses, as well as targeted preoperative support aimed at strengthening patients’ confidence, improving their understanding of perioperative information, and encouraging positive coping.

## Supplementary Information

Supplementary Material 1.

## Data Availability

Data supporting the findings of this study are available upon reasonable request from the corresponding authors. These data will not be made public owing to privacy and ethical restrictions.

## Abbreviations

VATS: Video-assisted thoracoscopic surgery
