# Supplementary material for: The relationship between preoperative anxiety, self-efficacy, and positive coping style in lung cancer patients undergoing video-assisted thoracoscopic surgery: a cross-sectional study
Source: BMC Psychol. 2026 Aug 10;14:1599. doi: 10.1186/s40359-026-05324-5 (PMC13737807; doi:10.1186/s40359-026-05324-5)
Supplement: Supplementary file 1 [file 40359_2026_5324_MOESM1_ESM.docx]

**Survey of Preoperative Anxiety, General Self-Efficacy, and Coping Styles**

**Patients with Lung Cancer Undergoing Video-Assisted Thoracoscopic Surgery (VATS)**

**Dear Participant,**

You are invited to participate in a survey examining factors related to preoperative anxiety. Your responses will provide important information for research on approaches to reducing anxiety before surgery. There are no right or wrong answers. Please select the response that best reflects your actual thoughts and experiences, answer every question, and avoid leaving any item blank. This questionnaire is for scientific research only, and the research team will protect your privacy. Completion of the questionnaire indicates that you have been informed about the survey and voluntarily agree to participate. Thank you for your valuable assistance.

# Section I. General Information

| **1. Sex** | ☐ Male ☐ Female |
| --- | --- |
| **2. Age** | ☐ 18–29 years ☐ 30–39 years ☐ 40–49 years ☐ ≥50 years |
| **3. Marital status** | ☐ Unmarried ☐ Married ☐ Divorced ☐ Widowed |
| **4. Ethnicity** | ☐ Han ☐ Ethnic minority |
| **5. Religious belief** | ☐ Yes ☐ No |
| **6. Education level** | ☐ Junior high school or below ☐ Technical secondary school or senior high school (including vocational high school) ☐ Associate degree ☐ Bachelor’s degree ☐ Master’s degree or above |
| **7. Place of residence** | ☐ Rural ☐ Urban |
| **8. Occupation** | ☐ Farmer ☐ Worker ☐ Civil servant ☐ Freelance/self-employed |
| **9. Current employment status** | ☐ Retired ☐ Unemployed ☐ Other |
| **10. Previous surgical history** | ☐ Yes ☐ No |
| **11. Payment method for medical expenses** | ☐ Provincial, municipal, or county urban medical insurance ☐ New Rural Cooperative Medical Scheme ☐ Self-pay ☐ Commercial insurance |
| **12. Monthly income** | ☐ <1,500 RMB ☐ 1,500–2,999 RMB ☐ 3,000–4,999 RMB ☐ ≥5,000 RMB |

# Section II. Preoperative Anxiety Assessment

**Instructions:** How anxious or concerned do you feel about each of the following at this moment? Read each item carefully and tick (✓) one box for each item.

| **Item** | **Never**  **(0)** | **Rarely**  **(1)** | **Sometimes**  **(2)** | **Often**  **(3)** | **Always**  **(4)** |
| --- | --- | --- | --- | --- | --- |
| 1. Risks associated with anesthesia during surgery. | ☐ 0 | ☐ 1 | ☐ 2 | ☐ 3 | ☐ 4 |
| 2. Doctors may discover other health problems during my surgery. | ☐ 0 | ☐ 1 | ☐ 2 | ☐ 3 | ☐ 4 |
| 3. Healthcare professionals may make mistakes during my surgery. | ☐ 0 | ☐ 1 | ☐ 2 | ☐ 3 | ☐ 4 |
| 4. Contracting an infection or becoming ill in the hospital environment. | ☐ 0 | ☐ 1 | ☐ 2 | ☐ 3 | ☐ 4 |
| 5. Being discharged before I have fully recovered. | ☐ 0 | ☐ 1 | ☐ 2 | ☐ 3 | ☐ 4 |
| 6. Experiencing pain or nausea after surgery. | ☐ 0 | ☐ 1 | ☐ 2 | ☐ 3 | ☐ 4 |
| 7. Costs associated with my surgery. | ☐ 0 | ☐ 1 | ☐ 2 | ☐ 3 | ☐ 4 |
| 8. Not receiving enough social support after surgery. | ☐ 0 | ☐ 1 | ☐ 2 | ☐ 3 | ☐ 4 |
| 9. Effects on my work or studies. | ☐ 0 | ☐ 1 | ☐ 2 | ☐ 3 | ☐ 4 |
| 10. My current health condition may complicate my surgery. | ☐ 0 | ☐ 1 | ☐ 2 | ☐ 3 | ☐ 4 |
| 11. How long it will take me to return to my normal daily activities or hobbies. | ☐ 0 | ☐ 1 | ☐ 2 | ☐ 3 | ☐ 4 |
| 12. Having no control over my anesthesia. | ☐ 0 | ☐ 1 | ☐ 2 | ☐ 3 | ☐ 4 |
| 13. Injections or intravenous infusions. | ☐ 0 | ☐ 1 | ☐ 2 | ☐ 3 | ☐ 4 |
| 14. My surgical wound. | ☐ 0 | ☐ 1 | ☐ 2 | ☐ 3 | ☐ 4 |
| 15. Unknown factors and risks during surgery. | ☐ 0 | ☐ 1 | ☐ 2 | ☐ 3 | ☐ 4 |
| 16. Remaining awake during surgery. | ☐ 0 | ☐ 1 | ☐ 2 | ☐ 3 | ☐ 4 |

# Section III. General Self-Efficacy Scale

**Instructions:** Read each statement carefully and tick (✓) the one response that best describes you.

| **Item** | **Not at all true (1)** | **Hardly true (2)** | **Moderately true (3)** | **Exactly true (4)** |
| --- | --- | --- | --- | --- |
| 1. I can always manage to solve difficult problems if I try hard enough. | ☐ 1 | ☐ 2 | ☐ 3 | ☐ 4 |
| 2. If someone opposes me, I can find the means and ways to get what I want. | ☐ 1 | ☐ 2 | ☐ 3 | ☐ 4 |
| 3. It is easy for me to stick to my aims and accomplish my goals. | ☐ 1 | ☐ 2 | ☐ 3 | ☐ 4 |
| 4. I am confident that I could deal efficiently with unexpected events. | ☐ 1 | ☐ 2 | ☐ 3 | ☐ 4 |
| 5. Thanks to my resourcefulness, I know how to handle unforeseen situations. | ☐ 1 | ☐ 2 | ☐ 3 | ☐ 4 |
| 6. I can solve most problems if I invest the necessary effort. | ☐ 1 | ☐ 2 | ☐ 3 | ☐ 4 |
| 7. I can remain calm when facing difficulties because I can rely on my coping abilities. | ☐ 1 | ☐ 2 | ☐ 3 | ☐ 4 |
| 8. When I am confronted with a problem, I can usually find several solutions. | ☐ 1 | ☐ 2 | ☐ 3 | ☐ 4 |
| 9. If I am in trouble, I can usually think of a solution. | ☐ 1 | ☐ 2 | ☐ 3 | ☐ 4 |
| 10. I can usually handle whatever comes my way. | ☐ 1 | ☐ 2 | ☐ 3 | ☐ 4 |

# **Section IV. Simplified Coping Style Questionnaire (SCSQ)**

**Instructions:** The following items describe attitudes and behaviours that people may adopt when experiencing setbacks or difficulties. Read each item carefully and tick (✓) the response that best reflects how often you use that approach: 0 = do not use, 1 = occasionally use, 2 = sometimes use, and 3 = often use.

| **Item** | **Do not use (0)** | **Occasionally use (1)** | **Sometimes use (2)** | **Often use (3)** |
| --- | --- | --- | --- | --- |
| 1. Distract myself through work, study, or other activities. | ☐ 0 | ☐ 1 | ☐ 2 | ☐ 3 |
| 2. Change my way of thinking and reconsider what is important in life. | ☐ 0 | ☐ 1 | ☐ 2 | ☐ 3 |
| 3. Try to see the positive side of things. | ☐ 0 | ☐ 1 | ☐ 2 | ☐ 3 |
| 4. Talk with others and share my worries. | ☐ 0 | ☐ 1 | ☐ 2 | ☐ 3 |
| 5. Avoid taking the problem too seriously. | ☐ 0 | ☐ 1 | ☐ 2 | ☐ 3 |
| 6. Stand my ground and strive for what I want. | ☐ 0 | ☐ 1 | ☐ 2 | ☐ 3 |
| 7. Identify several different ways to solve the problem. | ☐ 0 | ☐ 1 | ☐ 2 | ☐ 3 |
| 8. Seek advice from relatives, friends, or classmates. | ☐ 0 | ☐ 1 | ☐ 2 | ☐ 3 |
| 9. Change some of my previous practices or aspects of myself. | ☐ 0 | ☐ 1 | ☐ 2 | ☐ 3 |
| 10. Learn from how others handled similar difficult situations. | ☐ 0 | ☐ 1 | ☐ 2 | ☐ 3 |
| 11. Take up hobbies and actively participate in recreational or sports activities. | ☐ 0 | ☐ 1 | ☐ 2 | ☐ 3 |
| 12. Try to overcome my disappointment, regret, sadness, and anger. | ☐ 0 | ☐ 1 | ☐ 2 | ☐ 3 |
| 13. Try to rest or take a vacation and temporarily put the problem aside. | ☐ 0 | ☐ 1 | ☐ 2 | ☐ 3 |
| 14. Relieve distress by smoking, drinking alcohol, taking medication, or eating. | ☐ 0 | ☐ 1 | ☐ 2 | ☐ 3 |
| 15. Believe that time will change the situation and that all I can do is wait. | ☐ 0 | ☐ 1 | ☐ 2 | ☐ 3 |
| 16. Try to forget the whole situation. | ☐ 0 | ☐ 1 | ☐ 2 | ☐ 3 |
| 17. Rely on others to solve the problem. | ☐ 0 | ☐ 1 | ☐ 2 | ☐ 3 |
| 18. Accept the situation because there is no other option. | ☐ 0 | ☐ 1 | ☐ 2 | ☐ 3 |
| 19. Fantasize that a miracle may occur and change the situation. | ☐ 0 | ☐ 1 | ☐ 2 | ☐ 3 |
| 20. Comfort myself. | ☐ 0 | ☐ 1 | ☐ 2 | ☐ 3 |

**Thank you for completing the questionnaire.**
